# Spatio-temporal coherence of dengue, chikungunya and Zika outbreaks in Merida, Mexico

**DOI:** 10.1371/journal.pntd.0006298

**Published:** 2018-03-15

**Authors:** Donal Bisanzio, Felipe Dzul-Manzanilla, Hector Gomez-Dantés, Norma Pavia-Ruz, Thomas J. Hladish, Audrey Lenhart, Jorge Palacio-Vargas, Jesus F. González Roldan, Fabian Correa-Morales, Gustavo Sánchez-Tejeda, Pablo Kuri Morales, Pablo Manrique-Saide, Ira M. Longini, M. Elizabeth Halloran, Gonzalo M. Vazquez-Prokopec

**Affiliations:** 1 RTI International, Washington, DC, United States of America; 2 Centre for Tropical Diseases, Sacro Cuore-Don Calabria Hospital, Negrar, Verona, Italy; 3 Centro Nacional de Programas Preventivos y Control de Enfermedades (CENAPRECE) Secretaría de Salud Mexico, Mexico City, Mexico; 4 Health Systems Research Center, National Institute of Public Health, Cuernavaca, Mexico; 5 Centro de Investigaciones Regionales Hideyo Noguchi, Universidad Autonoma de Yucatan, Merida, Mexico; 6 Department of Biology, University of Florida, Gainesville, FL, United States of America; 7 Emerging Pathogens Institute, University of Florida, Gainesville, FL, United States of America; 8 Centers for Disease Control and Prevention, Atlanta, GA, United States of America; 9 Secretaria de Salud de Yucatan. Merida, Mexico; 10 Subsecretaría de Prevención y Promoción de la Salud, Mexico City, Mexico; 11 Unidad Colaborativa de Bioensayos Entomológicos, Campus de Ciencias Biológicas y Agropecuarias, Universidad Autónoma de Yucatán, Merida, Mexico; 12 Department of Biostatistics, University of Florida, Gainesville, FL, United States of America; 13 Center for Inference and Dynamics of Infectious Diseases, Seattle, WA, United States of America; 14 Vaccine and Infectious Disease Division, Fred Hutchinson Cancer Research Center, Seattle, WA, United States of America; 15 Department of Biostatistics, University of Washington, Seattle, WA, United States of America; 16 Department of Environmental Sciences, Emory University, Atlanta, GA, United States of America; University of Texas Medical Branch, UNITED STATES

## Abstract

Response to Zika virus (ZIKV) invasion in Brazil lagged a year from its estimated February 2014 introduction, and was triggered by the occurrence of severe congenital malformations. Dengue (DENV) and chikungunya (CHIKV) invasions tend to show similar response lags. We analyzed geo-coded symptomatic case reports from the city of Merida, Mexico, with the goal of assessing the utility of historical DENV data to infer CHIKV and ZIKV introduction and propagation. About 42% of the 40,028 DENV cases reported during 2008–2015 clustered in 27% of the city, and these clustering areas were where the first CHIKV and ZIKV cases were reported in 2015 and 2016, respectively. Furthermore, the three viruses had significant agreement in their spatio-temporal distribution (Kendall W>0.63; p<0.01). Longitudinal DENV data generated patterns indicative of the resulting introduction and transmission patterns of CHIKV and ZIKV, leading to important insights for the surveillance and targeted control to emerging *Aedes*-borne viruses.

## Author summary

Over the past decades, *Aedes*-borne viruses (dengue, chikungunya, Zika) have become a major source of morbidity within urban areas. Worldwide, public health response to these viruses is reactive to the occurrence of symptomatic cases (a small proportion of all infections). Here we used geocoded passive surveillance data to determine if areas of historically persistent dengue transmission fuel the introduction and propagation of other *Aedes*-borne viruses. This article provides quantitative evidence of the strong spatio-temporal overlap that occurs between dengue, chikungunya and Zika, all transmitted by *Aedes* aegypti mosquitoes in the city. Additionally, it emphasizes the value of analyzing long-term geo-coded passive surveillance information to help identify areas for prioritizing surveillance and control. Findings from this article open a window for considering historical DENV data to make predictions of likely sources of invasion for other emerging *Aedes*-borne viruses, as well as to the consideration of spatially-targeted approaches for delivery of vector control and surveillance. Arbovirus control in complex urban environments can greatly benefit from exploiting existing spatial information for better delivery of interventions.

## Introduction

The unprecedented global emergence of infectious disease threats has demonstrated how ill-prepared the world is to predict, rapidly respond to, and contain pandemics [1–3]. Zika virus (ZIKV), an emerging arthropod-borne flavivirus, has become a global public health threat since its emergence outside Africa in 2007 [4,5]. A large outbreak in French Polynesia in 2013–2014 showed that an otherwise clinically mild illness (common symptoms include mild fever, rash, arthralgia, and conjunctivitis) could also be responsible for severe neurological complications in adults (e.g., Guillain-Barre syndrome) [6,7]. Once active ZIKV transmission was recognized in Brazil in 2015–2016, the spectrum of disease severity expanded to include neurodegenerative complications in newborns (e.g., microcephaly) as well as other developmental sequelae grouped within the diagnostic term “congenital Zika syndrome” [4,8].

The World Health Organization declaration of Zika as a public health emergency of international concern on February 1, 2016, led to a global coordinated effort to improve vaccine development and scale-up vector control actions against the primary ZIKV vector in the Americas, *Aedes aegypti*. Failure in previous years to contain the transmission of chikungunya (CHIKV) and dengue (DENV) viruses, also primarily transmitted by *Ae*. *aegypti*, demonstrated the challenge in scaling-up vector control tools and interventions such as ultra-low volume (ULV) insecticide applications, larviciding and community mobilization, all with limited efficacy in preventing disease [9]. Although most DENV endemic countries in the Americas anticipated the arrival of ZIKV, they still performed only reactive interventions once symptomatic cases (assumed to be ~20% of all ZIKV infections, [4]) were detected. This led to significant delays in response and limited impact on virus containment, as seen in the rapid geographic spread of epidemics [5]. While some vaccine candidates may soon enter into Phase IIb/III trials [10], vector control and community education still remain the main preventive approaches to minimize the severe and devastating health outcomes associated with ZIKV infection.

DENV, CHIKV and ZIKV are all primarily transmitted by *Ae*. *aegypti* mosquitoes, leading to the early assumption that ZIKV would follow the path of the other two viruses in the Americas [11]. While this hypothesis is supported at regional scales, with the three viruses following similar transmission seasonality and regional circulation (e.g., [12]), it is unknown how their transmission pattern could differ within urban centers. DENV tends to show strong heterogeneity within cities, with some neighborhoods reporting higher sero-prevalence or transmission than others (e.g., [13–17]). Daytime human movement and aggregation patterns can influence exposure and are drivers of both human-*Ae*. *aegypti* contacts and rapid virus propagation, affecting DENV, CHIKV and ZIKV transmission [18–21]. Given the shared vector and ecology between the three viruses, we conducted spatio-temporal analyses on historical (2008–2015) DENV passive surveillance data and recent (2015–2016) CHIKV and ZIKV virus invasion events within the city of Merida, Mexico, and used the results to answer the following questions: Are DENV transmission hot-spots within cities also likely to be CHIKV and ZIKV transmission hot-spots? Do areas of historically persistent DENV transmission fuel the introduction and propagation of the other two viruses?

## Materials and methods

### Study site

The Yucatan Peninsula of Mexico is endemic for all dengue serotypes, with DENV1 and DENV2 dominating during 2008–2015 and DENV4 invading in 2013. Over the same period, 40,028 probable DENV cases have been reported from the city of Merida (S1 Text), the most important urban center (population ~1 million) and Yucatan State’s capital, and major contributor to the burden of virus transmission in the region [22]. Merida is located in a subtropical environment with mean temperatures ranging from 29°C in December to 34°C in July. The rainy season occurs from May to October and overlaps with the peak dengue transmission season between July and November, although cases occur year-round [23]. Dengue virus is widely distributed throughout the Yucatan peninsula, and the vector control strategies used by local authorities include ultra-low volume (ULV) spraying with the organophosphate insecticides chlorpyrifos and malathion and indoor space spraying with pyrethroids (deltamethrin) and organophosphates (malathion).

### Data sources and analysis

Recent operational innovations in the surveillance and control of *Aedes*-borne viruses in Mexico led to the development of a comprehensive online system for the capture, visualization and analysis of entomologic and epidemiologic data [24]. The National System of Epidemiological Surveillance of the Mexican Ministry of Health (operational since 2008) includes a nationwide, web-based, geographic information system where disease-specific reported DENV, CHIKV and ZIKV cases are automatically geocoded and mapped at the geographic scale of households [24]. The following information was obtained for the city of Merida: probable and laboratory confirmed cases geocoded to the household level and with information of age, epidemiologic week of the onset of symptoms for every case; disease infection status (DENV, CHIKV, ZIKV) for each case; DENV serotype (for a subset of laboratory-confirmed cases). Demographic information was obtained for the city of Merida (2010 census) from Mexico’s National Census and aggregated at the level of census tracts, called Area Geoestadistica Basica (AGEB). Tracts aggregate up to 50 city blocks (average area, 0.5 km^2^; Standard Deviation, 0.3 km^2^), and Merida has 540 tracts.

Epidemiologic information was aggregated at the spatial scale of census tracts and the temporal scale of weeks, with separate datasets generated for each virus. The weekly count of cases was normalized, to allow for comparisons between years and viruses, by calculating, for each virus, weekly z-scores per tract as follows:

$$z_{i}=\frac{N_{i}-\mu }{\sigma }$$ (1)

where *N*_*i*_ is the number of cases in tract *i*, *μ* the mean case count for the city, and σ the standard deviation of case counts for the city. For a given week, the obtained z-score was rescaled dividing it by census tract’s maximum value of case counts.

The weekly trend in DENV cases was analyzed to separate, during the years 2008–2015, the epidemic from non-epidemic transmission periods of the time-series using change point detection methods [25]. A G* local spatial clustering test [26] was applied to annual normalized case counts separately for epidemic and non-epidemic periods (for DENV) and by year for CHIKV and ZIKV. The significance of the computed G* was evaluated by comparing observed values to the null hypothesis of random case distribution by randomly re-assigning the tract labels to the case counts. This was performed using 100,000 Monte Carlo randomizations. Tracts with statistically significant (p<0.05, calculated adopting Bonferroni correction) high z-scores, defined as hot-spots, were mapped for each virus and epidemic period (DENV) or year (CHIKV, ZIKV). For DENV, G* output was aggregated to estimate a count of time-periods that a tract was identified as a hot-spot.

A Kendall *W* test evaluated the spatio-temporal overlap between DENV, CHIKV and ZIKV z-values by tract. *W* measures concordance between two datasets, ranging from +1 (complete agreement) to -1(no agreement) and was calculated for all combinations of virus pairs (DENV-CHIKV, DENV-ZIKV, CHIKV-ZIKV). A second approach to evaluate spatio-temporal overlap between viruses consisted in overlaying the persistence of DENV hot-spots per tract with the hot-spots identified for CHIKV (2015, 2016) and for ZIKV (2016).

Data from a longitudinal DENV cohort study following 1,666 residents aged 1–60 from Merida was utilized to contrast DENV seroprevalence of those living in areas identified as hot-spots versus those living outside. Briefly, 17 elementary schools distributed across Merida in areas thought to differ in DENV incidence (low incidence in the north and high incidence in the south) were chosen as the hubs for participant enrollment and follow-up. From the school student list, 464 children were selected (up to 50 per grade per school) for the serological follow-up, which also included all family members living in the same residence and who consented to the study. Annual blood draws were performed in 2015 and positivity to any DENV serotype was assessed by Yucatan State Laboratories. Prior exposure to dengue and age-specific serostatus were determined using Panbio IgG indirect ELISA. Standard cut-off points were used for defining positive (≥ 12 Panbio units) from negative samples (<9 Panbio units) and indeterminate for those in between. From the people enrolled in the cohort, we selected those whose residence was found inside the areas identified as DENV hot-spots and randomly selected a similar number of individuals enrolled in the cohort living outside the area. As our retrospective analysis covered the period 2008–2015, we focused our analysis on children aged 8 years or younger. A mixed-effects logistic regression model quantified the odds ratio for the following fixed effects: DENV cluster (i.e., living outside or inside the 2008–2015 DENV hot-spot areas) and age. A random intercept was included at the level of the household to account for the occurrence of nested samples within each house. Models were run with the R package lme4 [27].

### Ethics statement

Protocols for processing and analyzing data were approved by Emory University’s ethics committee under protocol ID: IRB00088659. The protocol was also approved by the Ethics and Research Committee from the O'Horan General Hospital from the state Ministry of Health, Register No. CEI-0-34-1-14. All cases were anonymized before being analyzed.

## Results

### Spatio-temporal dynamics of DENV transmission

A total of 40,028 clinically apparent DENV cases were reported to the public health system of Merida during 2008–2015, 94.5% (37,894) occurring in eight epidemic periods lasting on average 34 weeks (range: 24–44) (Fig A in S1 Text). Approximately 30% of all cases were confirmed by laboratory assays (IgM and PCR). During this period, three DENV serotypes circulated in Merida; DENV1 predominated in most years, DENV2 invaded in 2009 and DENV4 in 2013 (Fig B in S1 Text). About 40% of reported cases occurred in <15 year olds, with the proportion of cases by age-class not differing significantly between years (Fig C in S1 Text) (Fisher’s exact test, p < 0.05).

**Fig 1:**
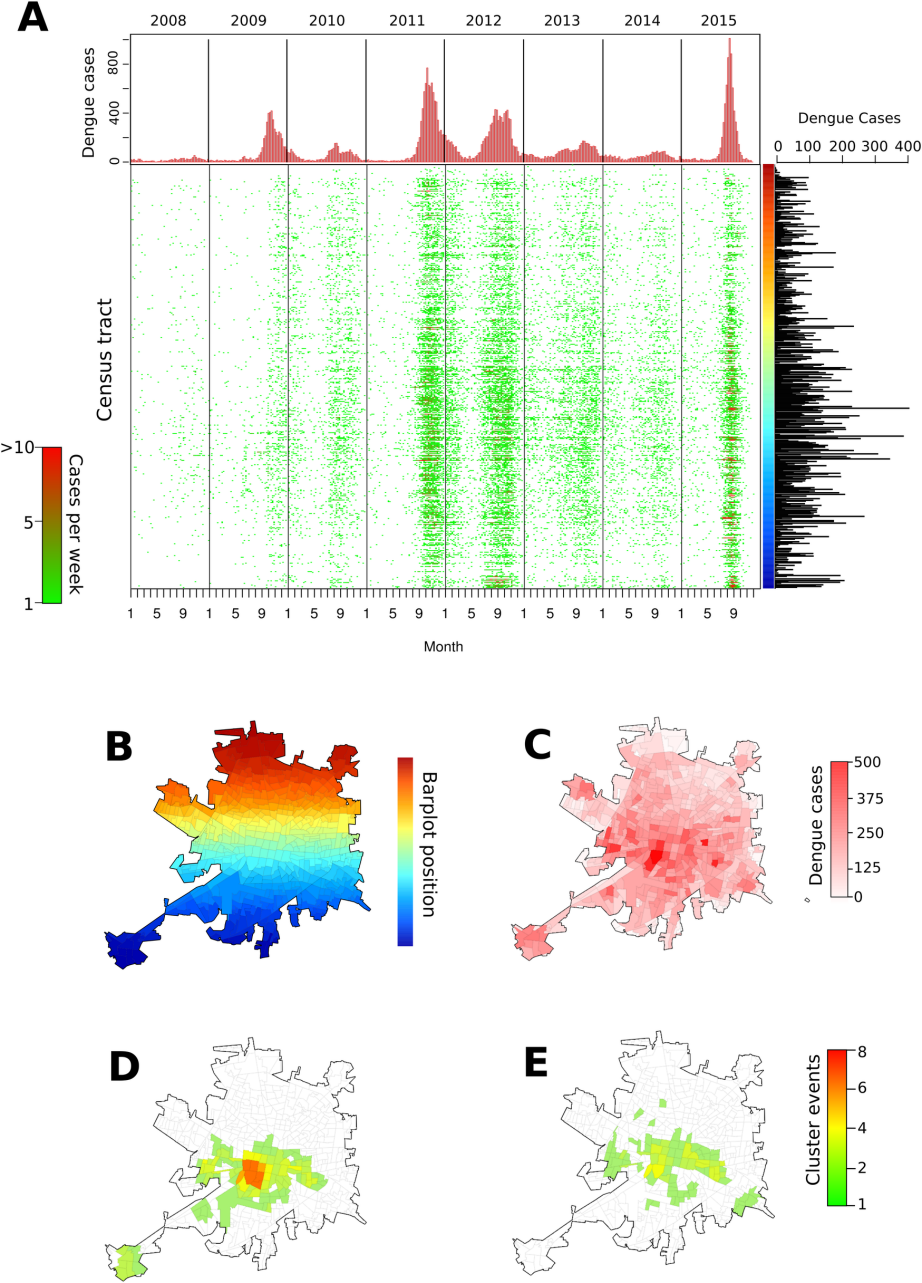
Spatio-temporal distribution of standardized dengue virus (DENV) cases in Merida, Mexico, during 2008–2015. (A) Weekly case counts are shown as bar plot (above) and by census tract (each row is a tract and rows are arranged by geographic proximity, with near units being next to each other, as shown in the color bar located on the right margin which matches the color map in (B)). Right panel shows total count of dengue cases per census unit during 2008–2015, which is mapped in (C). Panel (D) shows the frequency of outbreak periods in which each census unit was found to be a statistically significant hot-spot of DENV transmission. Panel (E) shows the same information for non-outbreak periods. Source of the census tract boundaries was Instituto Nacional de Estadística y Geografía (INEGI), 2010.

The weekly spatio-temporal progression of standardized DENV case counts within the city was heterogeneous across census tracts, showing a complex pattern of emergence and persistence over the 8-year period (Fig 1A and 1B, Fig D in S1 Text). When summarized over the entire period, standardized case counts aggregated in nearby tracts (Fig 1C) suggested the occurrence of strong autocorrelation. The Getis-Ord G* statistic, calculated separately for epidemic and non-epidemic periods (Fig E and Fig F in S1 Text), identified tracts within Merida with persistent DENV transmission up to 6 years (epidemic, Fig 1D) and 4 years (non-epidemic, Fig 1E) concentrated in the center of the city. Altogether, the hot-spot areas contained 41.9% (16,773) of all reported symptomatic cases within 27.8% of the city’s 540 census tracts.

**Fig 2:**
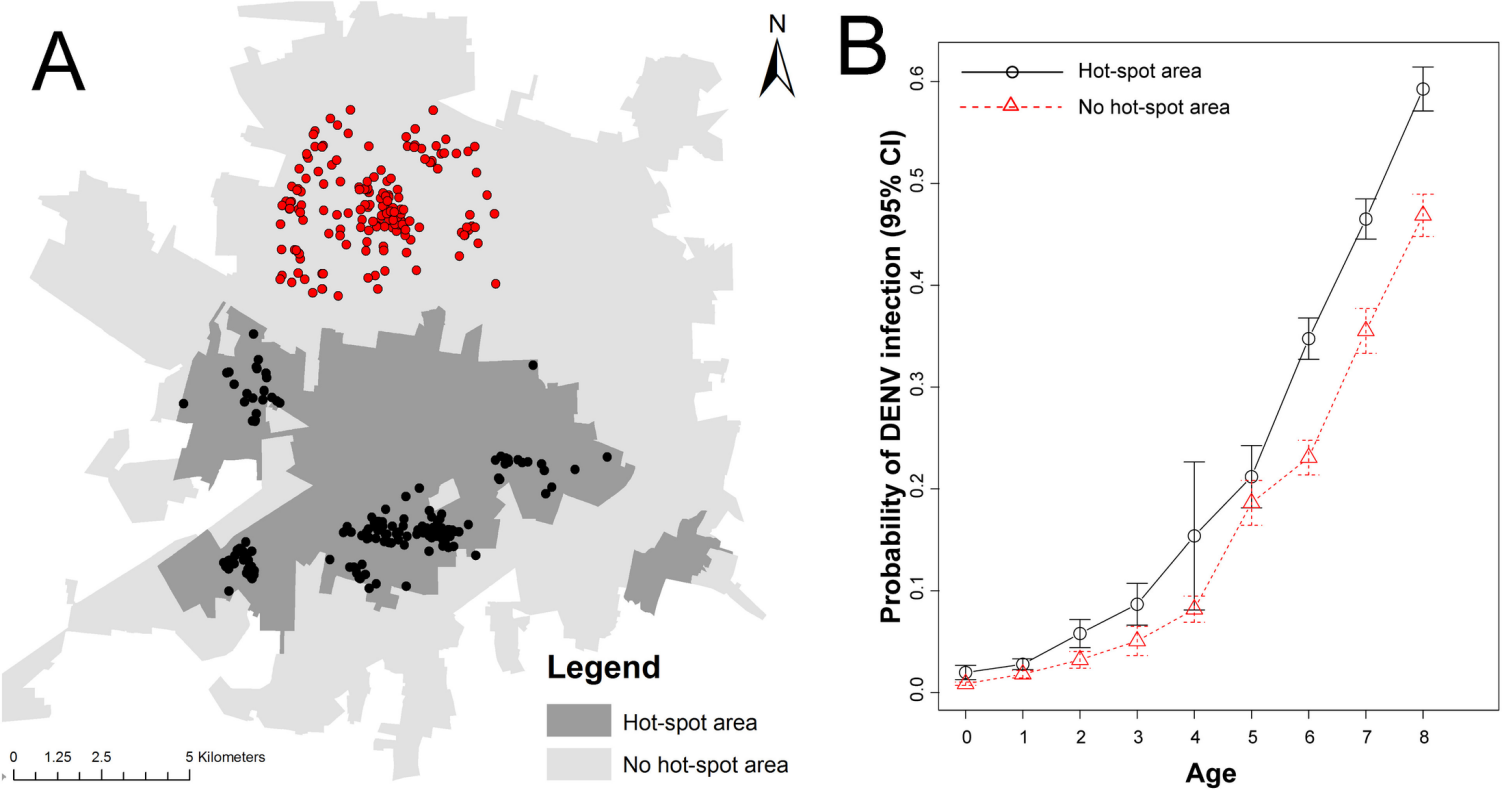
Validation of DENV spatial clustering. (A) location of the residence of 505 children aged 8 or younger and who provided a blood sample in 2015, stratified by those residing inside (black) and outside (red) the 2008–2015 DENV hot-spot area. (B) Average DENV infection probability, estimated from a mixed-effects logistic regression model, for the children living inside (black solid line) or outside (red dotted line) the 2008–2015 DENV hot-spot area. Error bars indicate the standard error of the mean. Details of the model are shown in Table 1. Source of the census tract boundaries was INEGI, 2010.

Serological status for DENV infection in 505 children aged 8 or younger belonging to a longitudinal cohort study was compared between those residing inside and outside the hot-spot areas (244 children inside and 261 children outside the area) (Fig 2A). Living inside the hot-spot area was associated with a significantly higher infection probability than living outside (odds ratio, 1.71 [95%CI, 1.08–2.20]; p < 0.05), after adjusting for the age of the child and the occurrence of nested cases within households (Table 1). Model-predicted DENV infection probabilities were significantly higher in children aged 6–8 years living inside a hot-spot area, compared to children the same age living outside (Fig 2B).

**Table 1:** Odds ratio estimated from a mixed-effects logistic regression model evaluating the association between DENV seroprevalence in cohort participants aged 8 or younger and their location of residence (2008–2015 DENV clustering area).

	Odds Ratio				
Parameter	Estimate	2.5%	97.5%	Z-value	P-value
DENV Cluster*	1.710	1.078	2.861	2.199	0.0279
Age**	1.739	1.471	2.133	5.918	<0.001
Intercept	0.010	0.002	0.036	-6.596	<0.001

* Residence of participant found outside the 2008–2015 DENV clustering area was used as reference.

** Age at the time of providing a blood sample.

### Association with CHIKV and ZIKV transmission

In 2015, the first CHIKV case was reported in a tract within the persistent DENV clustering area (Fig G in S1 Text). From this tract, the virus propagated rapidly throughout the city (counting 1,101 probable cases) (Fig G in S1 Text), leading to difficulties in containing both DENV and CHIKV. ZIKV was first reported in April 2016, also inside the persistent DENV clustering area (Fig G in S1 Text). The 2016 ZIKV outbreak included 2,273 reported cases (of which ~50% were in <15 year olds, Fig H in S1 Text). No Zika-related microcephaly cases were yet detected in newborns to mothers infected during this first wave of ZIKV invasion. The tracts where the first 10 CHIKV and ZIKV cases were reported overlapped 100% and 75%, respectively, with the DENV persistent clustering area (Fig G in S1 Text).

**Fig 3:**
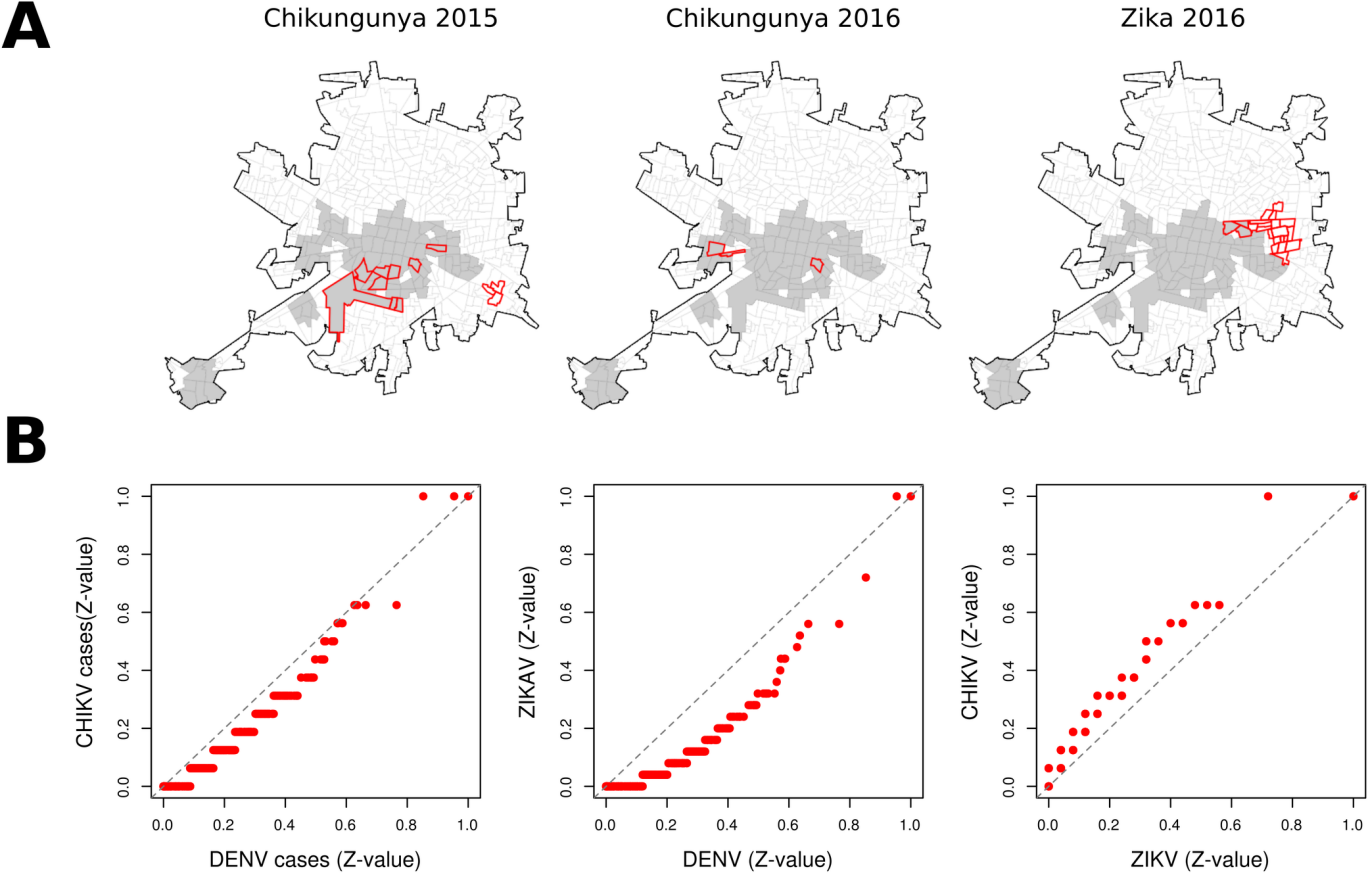
Spatial pattern of arbovirus hot-spots in Merida. (A) Hot-spots of CHIKV (2015–2016) and ZIKV (2016) standardized case counts overlapped with persistent DENV clustering area (gray polygons represent persistent DENV clustering areas and red polygons represent statistically significant yearly hot-spots for CHIKV and ZIKV). (B) Q-Q plots comparing distribution of standardized case counts of DENV, CHIKV, and ZIKV. Source of the census tract boundaries was INEGI, 2010.

The DENV, CHIKV and ZIKV case counts by tract showed a significantly (p<0.01) high coherence, with Kendall’s W values of 0.75 for DENV-ZIKV comparisons, 0.72 for DENV-CHIKV and 0.63 for CHIKV-ZIKV. The high similarity among DENV, CHIKV, and ZIKV are also evident when comparing distribution of standardized case counts using quantile-quantile (Q-Q) plots (Fig 3A). A 35.6% (392) of all reported CHIKV cases and 66.9% (1,509) of all ZIKV cases were found within the area of persistent DENV clustering. When comparing the location and extent of hot-spots, 9 (64.3%) CHIKV clusters for 2015 and 2016 fully overlapped with the 2008–2015 DENV clustering, and 3 (14.3%) clusters of high ZIKV standardized case counts overlapped with the DENV clustering area (Fig 3A).

## Discussion

Combining powerful analytic methods with detailed retrospective data provides evidence of the significant within-city spatio-temporal overlap between three *Aedes*-borne viruses. While forecasting the exact timing and introduction points of CHIKV and ZIKV within a city would be bound with significant uncertainty [28], historical spatio-temporal DENV surveillance data can be analyzed to identify the extent of high transmission areas that would serve as important transmission foci for novel *Aedes*-borne viruses.

Surveillance data has characterized the temporal pattern of CHIKV and ZIKV invasion in areas with active DENV transmission in great detail (e.g., [6,12]). Such descriptive studies provide evidence of high temporal overlap between the three viruses, with transmission peaks of CHIKV and ZIKV occurring during the same time periods as DENV peaks. In Merida, the three viruses not only overlapped in time, but they also exhibited strong spatio-temporal coherence in their distribution. The first CHIKV and ZIKV cases occurred within the DENV clustering area with a strong match between pathogens. It is important to note the partial mismatch between ZIKV and DENV hot-spots. While stochasticity in the initial virus introduction point may contribute to this finding, it is worth mentioning that in 2016 the Yucatan Ministry of Health intensified vector control interventions (i.e., indoor space spraying, ULV spraying and the use of larvicide) in the same areas predicted as hot-spots (based on our preliminary maps) as a way to potentially slow-down the spread of the virus. Whether this enhanced control contributed to the reduced frequency of ZIKV clustering in DENV hot-spots (even in the early presence of cases in the area) will require further study.

Mathematical models predict that, for pathogens that show strong spatial or social heterogeneity, targeted interventions would represent a more effective means of disease mitigation as compared to blanket control [29,30]. Focusing efforts in high transmission areas has the additional benefits of increased efficiency by better allocating limited personnel and resources [31]. Recognizing areas of persistent DENV, CHIKV or ZIKV transmission can enable the pre-emptive deployment of vector control before transmission is apparent [32]. The possible benefits of such pro-active, targeted control may actually be experienced on a larger spatial scale, as interventions may additionally protect individuals who visit the hot-spot areas but do not reside in them [33]. Thus, in settings where *Aedes*-borne diseases are seasonal, targeting vector control to traditional hot-spot areas in an anticipatory fashion during the period of low transmission can lead to efficiencies in intervention coverage (when personnel workload is at its lowest) and theoretically provide the highest intervention effectiveness (~60–90% of cases prevented) [34]. Alternatively, vector habitat modification strategies such as housing improvement [35,36] may be more cost-effective and justifiable if undertaken within hot-spots. Before targeted control can be broadly recommended, our findings will have to be validated in other urban centers (ideally, of differing size and level of DENV endemicity), and the epidemiological impact of targeting hot-spots will need to be empirically evaluated and weighed against the value of reactive interventions. Additionally, any targeted intervention will have to be monitored for potential future changes in epidemiological trends, like the emergence of new hot-spots in the city due to shifts vector abundance or socio-environmental conditions.

Several issues limit the quality and power of passive surveillance data and the potential for their use to forecast DENV transmission [28,37]. The data do not capture the disproportionately large number of infections that occur sub-clinically or that are mild enough not to result in a visit to a doctor. In many areas, DENV may be misdiagnosed as other febrile illnesses (e.g., influenza, leptospirosis [38]), adding uncertainty to observed trends. Additionally, given *Ae*. *aegypti* is a day-biting mosquito, mapping the place of residence may not correspond to the location where infection occurred [18,39]. Finally, in cities where *Ae*. *aegypti* and *Ae*. *albopictus* co-inhabit, spatial patterns of arbovirus transmission may be potentially influenced by their apparent disjoint distribution within urban areas (e.g., [40]). In Merida, where *Ae*. *aegypti* is the only vector and *Ae*. *albopictus* is not present, aggregating case data to relatively small (up to 50 city blocks) geographic units and analyzing standardized data over multiple years provided a strong signature of the spatial pattern of DENV circulation. About 27.8% (150) of the city districts accounted for 41.9% of cases, and such transmission heterogeneity was independently captured epidemiologically in the age-dependent sero-prevalence of DENV infection in children aged 8 years or younger. The ability of spatial clustering tests performed on passive surveillance data to capture strong trends in virus sero-prevalence provide a prospect for the development of city-wide risk maps, particularly if analyses involve multiple years and several virus introduction events.

Several spatial epidemiology studies have determined the set of census-derived environmental and socio-economic variables associated with DENV hot-spots. For instance, in Machala (Ecuador) hot-spots of dengue incidence at the level of census tracts was associated with older age and female gender of the head of the household, greater access to piped water in the home, poor housing condition, and less distance to the central hospital ([41]). In an endemic city in Venezuela, DENV hot-spots at the block level were associated with living in crowded conditions, having an occupation of domestic worker/housewife and not using certain preventive measures against mosquitoes ([42]). The contrast between these and other spatial epidemiology studies point to an important issue related to the finding of a generalized explanation to the elevated risk of DENV infection in hot-spots. Census variables vary from country to country, studies use data at different levels of aggregation, many census variables are likely auto-correlated, and there is an almost absence of entomological information. Thus, our study excluded imputing hot-spots to specific census variables because we will be performing a comprehensive environmental and anthropological assessment of the root drivers of the epidemiological trends observed in Merida.

We acknowledge several limitations to our study. While we used residential addresses in our analyses, we are aware that this excludes the potential for infection in areas other than the home [18,21]. Thus, our study was not able to explicitly account for movement and exposure in locations other than the residence, which may have obscured both the passive surveillance and the serological data. Future human mobility studies in Merida will help elucidate the role of hot-spots as foci of infection from both their residents and visitors from other parts of the city. The lack of Zika-related microcephaly cases in Merida at the point of analysis prevented evaluating any possible overlap between them and the historical DENV hot-spot areas. Given the possibility for immune interaction between viruses ([43]), evaluating if areas of intense DENV transmission lead to higher risk of microcephaly in newborns can lead to important insights for disease surveillance and control. While laboratory diagnosis of all three viruses is performed routinely in Mexico, current policy dictates that testing should be reduced once outbreaks are declared. This prompted us to include in our analyses clinically diagnosed (i.e., without laboratory confirmation) cases in addition to those with a laboratory confirmation.

While vaccines are seen as an ultimate disease mitigation strategy, their development can lag years after pathogen emergence [44], leaving case management and outbreak containment as the first line of defense. Thus, public health efforts face a challenging prospect: identifying vulnerable populations or likely transmission hot-spots as an approach to pro-actively mitigate emerging pathogen introduction and propagation. Recognizing the critical need to improve surveillance to prevent and rapidly contain pathogen transmission, the World Health Organization (WHO) is developing a global coordination mechanism for the containment of emerging pathogens [3]. Crucial to such effort is the integration of surveillance data and robust analytical methods to anticipate and better respond to disease threats [45,46]. Leveraging historical DENV, CHIKV and ZIKV data can help endemic countries improve control and surveillance programs by acknowledging and accounting for inherent spatial variability in risk within urban areas. Downscaling existing global risk maps (e.g., [47,48]) to capture within-city transmission risk is an important next step in disease mapping, as this can provide policy-makers with a more precise tool for intervention planning and strategic deployment at an operational scale.

## Supporting information

S1 Text Supporting figures showing: A) weekly count of DENV symptomatic cases reported to the public health system; B) relative frequency of isolated DENV serotypes; C) age-structure of reported DENV cases; D) standardized DENV case counts by year and census tract; E and F) results from the Getis-Ord G* statistic test; G) time series of weekly case counts for CHIKV (A) and ZIKV (B) in Merida; H) age-structure of reported CHIKV and ZIKV.
(PDF)
