# Supplementary material for: Spatio-temporal coherence of dengue, chikungunya and Zika outbreaks in Merida, Mexico
Source: PLoS Negl Trop Dis. 2018 Mar 15;12(3):e0006298. doi: 10.1371/journal.pntd.0006298 (PMC5870998; doi:10.1371/journal.pntd.0006298)

Supplementary Figures

Figure A. **Weekly count of DENV symptomatic cases reported to the public health system of Merida, Yucatan.** Gray boxes indicate epidemic periods (roman numbers on top), while white boxes indicate non-epidemic periods. Red lines point to the beginning of an epidemic period and green lines indicate the end of the same period. Dashed lines indicate the beginning and end of each calendar year.

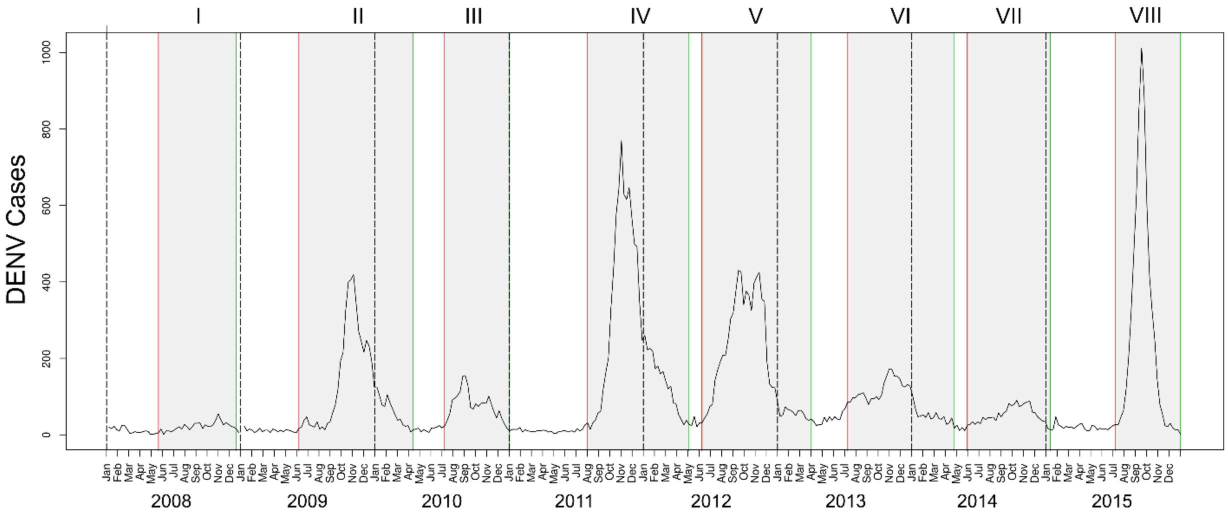

Figure B. Relative Frequency of DENV serotypes isolated in Merida, Yucatan, during 2008-2015.

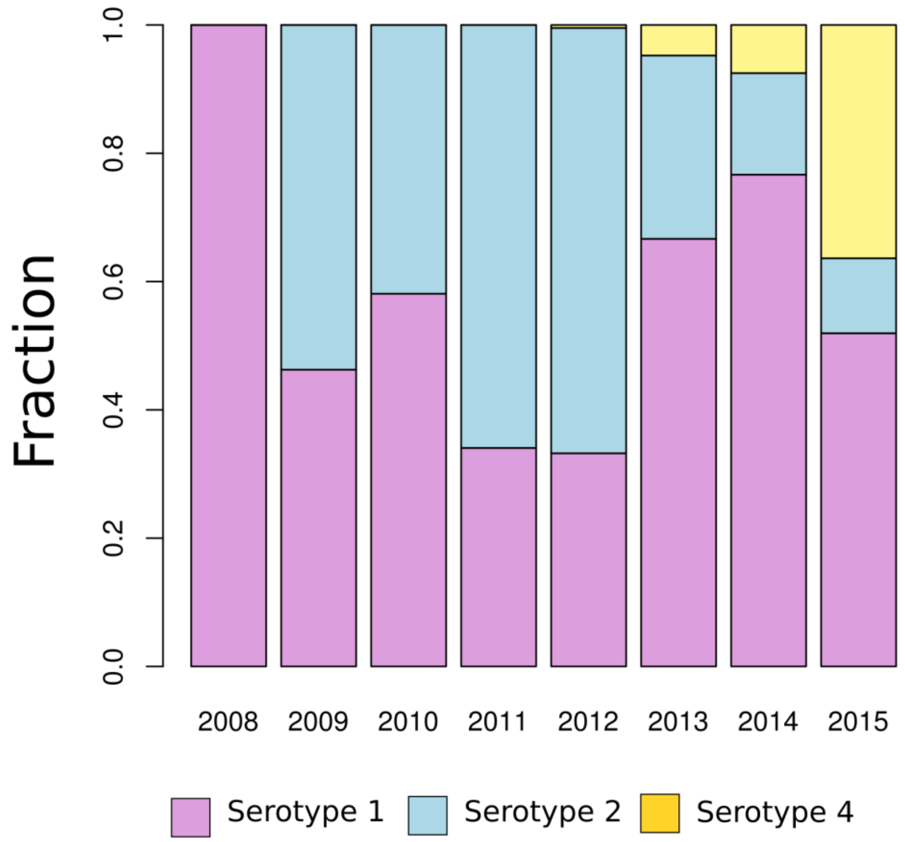

Figure C: Age-structure of reported DENV cases in the city of Merida, Mexico, for 2008-2015.

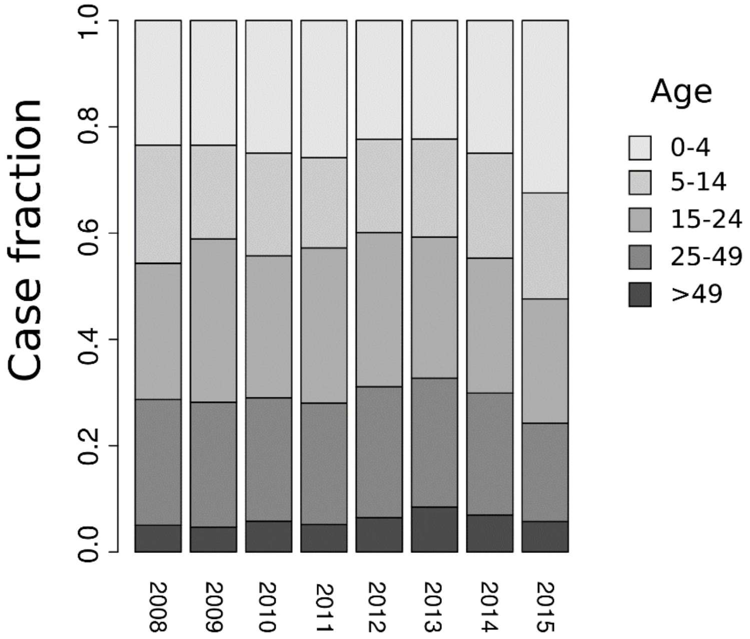

Figure D. Standardized DENV case counts by year and census tract for Merida, 2008-2015.

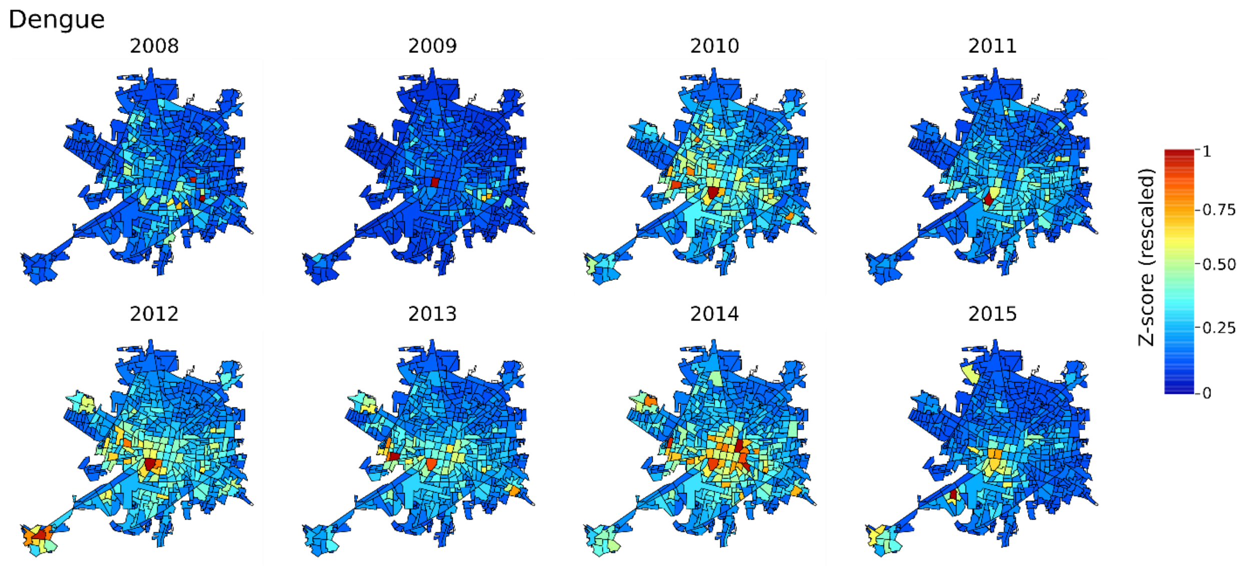

Figure E. Results from the Getis-Ord *G statistic test identifying tracts with statistically significant (p<0.05) high standardized case counts for outbreak periods (red polygons).** Map in lower right shows cumulative count of periods in which a tract was identified as a hot-spot.

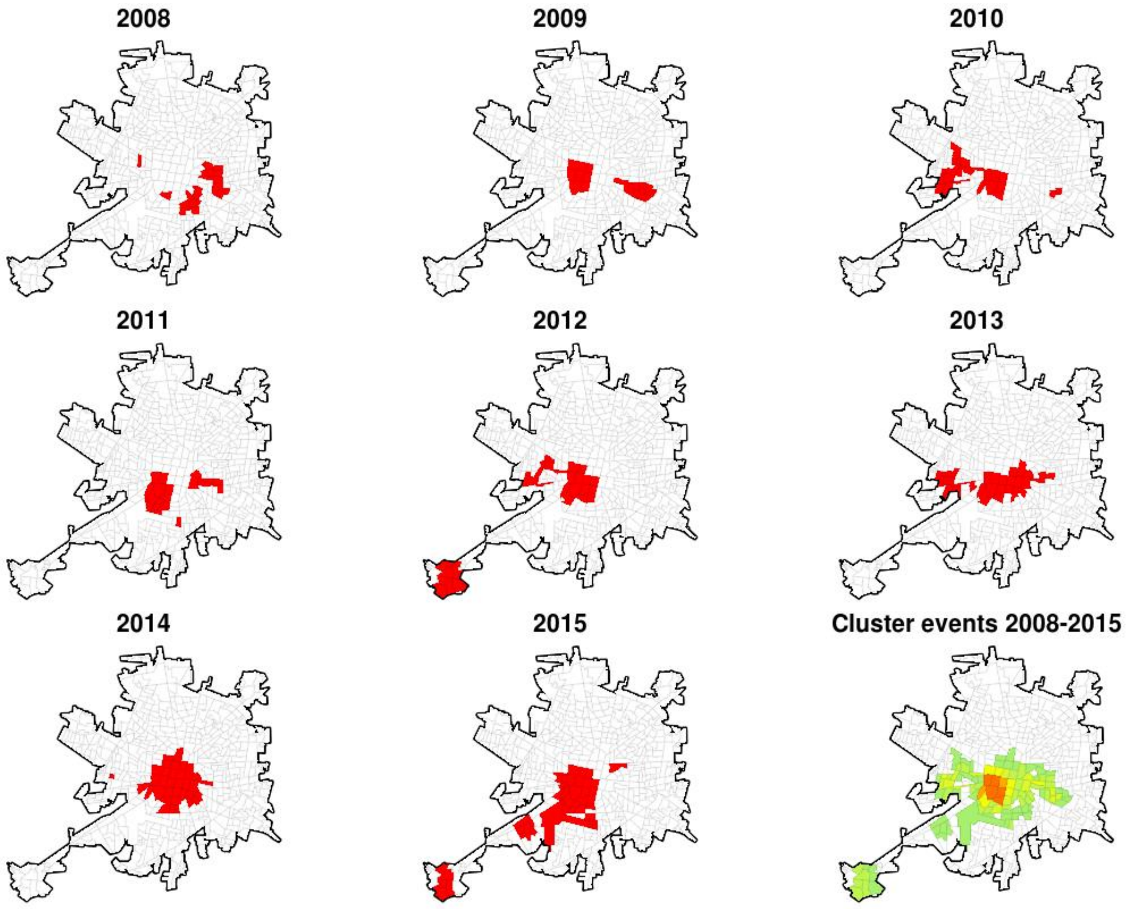

Figure F. Results from the Getis-Ord *G statistic test identifying tracts with statistically significant (P<0.05) high standardized case counts for non-outbreak periods (red polygons).** Map in lower right shows cumulative count of periods in which a tract was identified as a hot-spot.

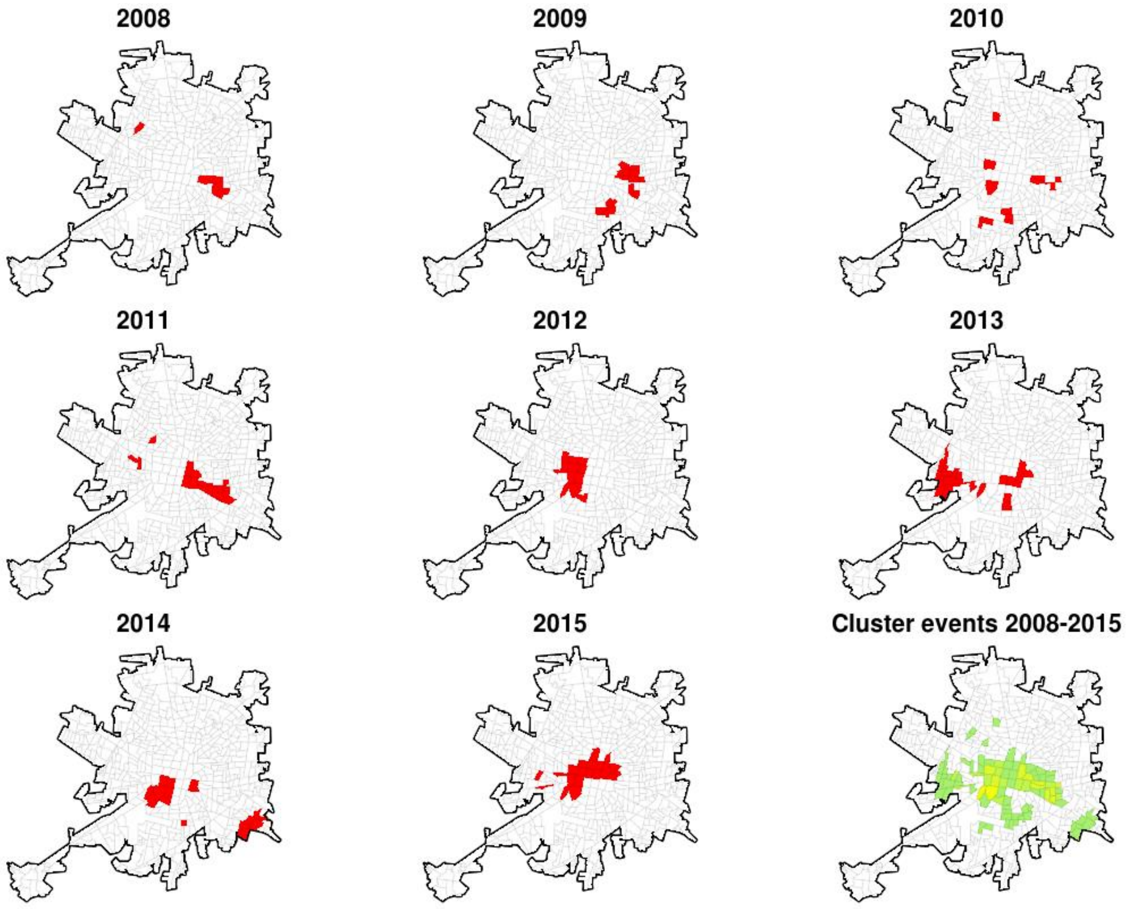

Figure G. **Time series of weekly case counts for CHIKV (A) and ZIKV (B) in Merida, Mexico.** Location of tracts where the first (green) and the first 10 (red) cases of CHIKV (C) and ZIKV (D) were reported. Gray shade highlights the persistent 2008-2015 DENV clustering area of the epidemic periods.

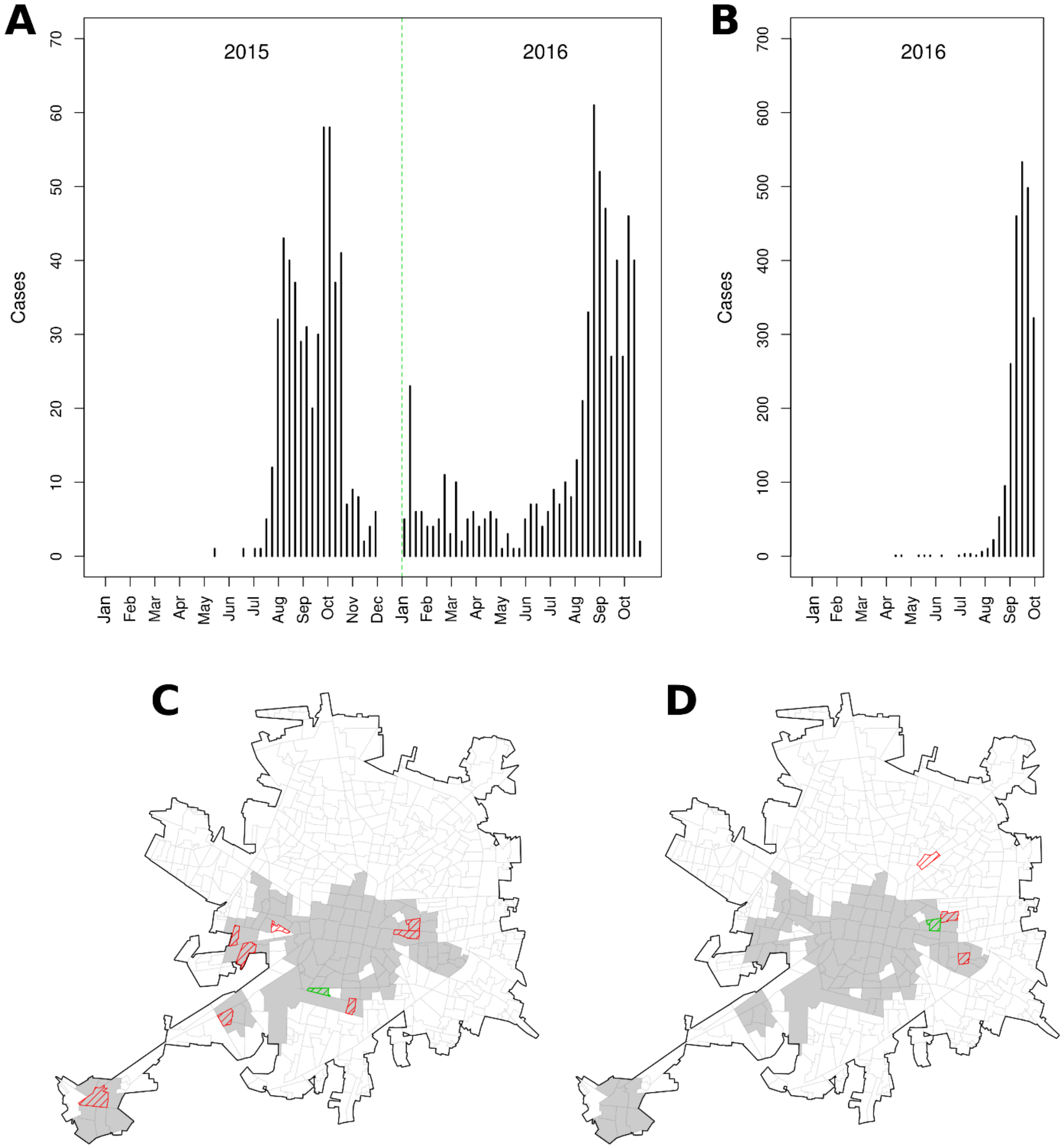
A
2015
2016
Cases
0
10
20
30
40
50
60
70
Jan
Feb
Mar
Apr
May
Jun
Jul
Aug
Sep
Oct
Nov
Dec
Jan
Feb
Mar
Apr
May
Jun
Jul
Aug
Sep
Oct
B
2016
Cases
0
100
200
300
400
500
600
700
Jan
Feb
Mar
Apr
May
Jun
Jul
Aug
Sep
Oct
C
D

Figure H. Age-structure of reported CHIKV and ZIKV cases in the city of Merida, Mexico, for 2015-2016.

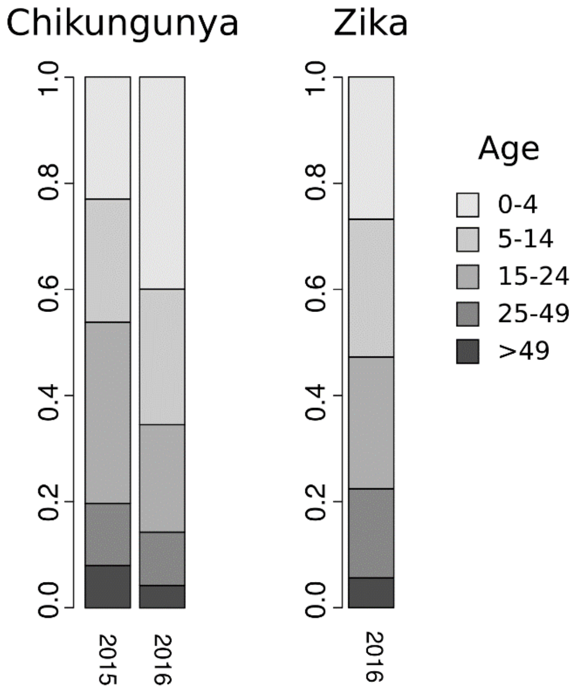

Supplement: S1 Text — Supporting figures showing: A) weekly count of DENV symptomatic cases reported to the public health system; B) relative frequency of isolated DENV serotypes; C) age-structure of reported DENV cases; D) standardized DENV case counts by year and census tract; E and F) results from the Getis-Ord G* statistic test; G) time series of weekly case counts for CHIKV (A) and ZIKV (B) in Merida; H) age-structure of reported CHIKV and ZIKV. (PDF) [file pntd.0006298.s001.pdf]
